# Non‐specific interstitial pneumonia associated with clinically amyopathic dermatomyositis showing “crazy paving” appearance on thin‐section lung CT

**DOI:** 10.1002/rcr2.326

**Published:** 2018-05-03

**Authors:** Yuya Aono, Tatsuru Eifuku, Tomohiro Uto, Jun Sato, Shiro Imokawa, Takafumi Suda

**Affiliations:** ^1^ Department of Respiratory Medicine Iwata City Hospital Iwata Japan; ^2^ Second Division, Department of Internal Medicine Hamamatsu University School of Medicine Hamamatsu Japan

**Keywords:** Autoimmune disease, collagen vascular diseases, interstitial lung disease, radiology and other imaging

## Abstract

The “crazy paving” appearance consists of ground‐glass opacity superimposed on a network of linear opacities on thin‐section computed tomography (CT) images of the lung. This finding has been described in a variety of diseases but is extremely rare in patients with non‐specific interstitial pneumonia (NSIP). We describe a 45‐year‐old woman with biopsy‐proven NSIP associated with clinically amyopathic dermatomyositis that showed a “crazy paving” appearance on thin‐section CT of the lung. Clinicians should include NSIP in the differential diagnosis in patients presenting with “crazy paving” appearance on thin‐section chest CT.

## Introduction

The “crazy paving” appearance on thin‐section computed tomography (CT) of the lung comprises ground‐glass opacity superimposed on a network of linear opacities. It has been described in a variety of diseases but is extremely rare in patients with non‐specific interstitial pneumonia (NSIP). We describe a 45‐year‐old woman with biopsy‐proven NSIP associated with clinically amyopathic dermatomyositis (CADM) that showed a “crazy paving” appearance on thin‐section CT of the lung.

## Case Report

A 45‐year‐old woman was referred to our hospital for investigation of a chest X‐ray abnormality detected at a routine medical check‐up. She was a former smoker (2 pack‐year). She had no history of occupational dust exposure and denied fever, shortness of breath, and cough. There were no signs of arthralgia, muscle ache, or weakness. She had dysmenorrhea and had been treated with a hormone preparation for more than a year. Physical examination showed mechanic’s hands on both fingers, Gottron’s papules on the dorsa of the knuckles, and keratotic erythema on her elbows and knees. No crackles were audible on auscultation.

Laboratory findings revealed a white blood cell count of 6700/μL, C‐reactive protein 0.00 mg/dL, creatine kinase (CK) 107 U/L, lactate dehydrogenase 233 U/L, KL‐6 2990 U/mL, and SP‐D 507 ng/mL. Screening for anti‐aminoacyl tRNA synthetase (ARS) antibodies was positive, but other autoimmune antibodies, including anti‐Jo‐1 antibody and anti‐MDA‐5 antibody, were negative. Spirometry and diffusing capacity for carbon monoxide were within normal reference ranges.

**Figure 1:**
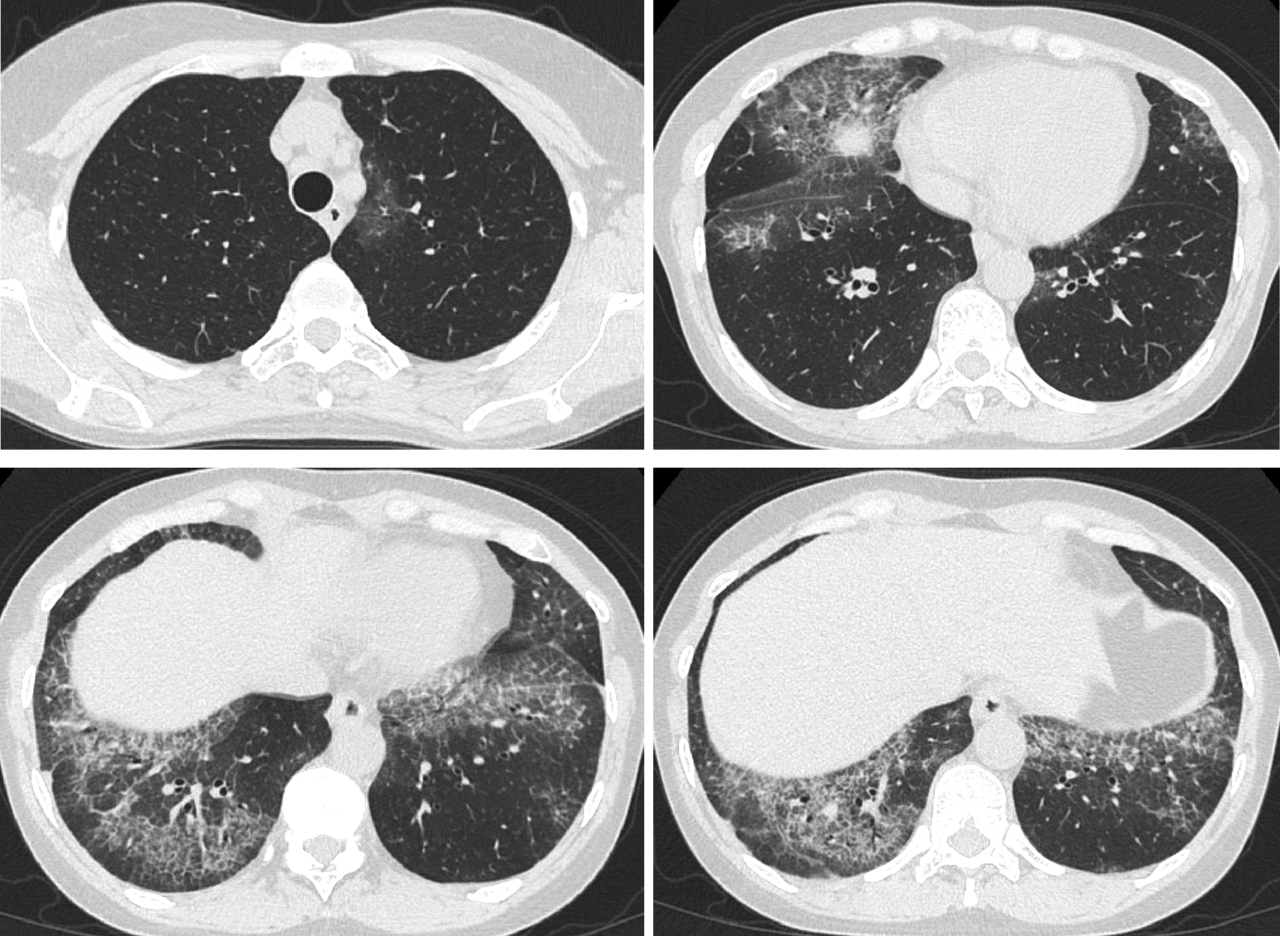
Thin‐section computed tomography of the lung showing bilateral patchy ground‐glass opacifications with interlobular septal thickening and intralobular lines, predominantly in the lower lobes (“crazy paving” appearance).

Chest X‐ray showed bilateral consolidation, predominantly in the lower lung fields, associated with volume loss of the lower lobes. Chest CT showed bilateral patchy ground‐glass opacifications with interlobular septal thickening and intralobular lines, referred to as “crazy paving” appearance, predominantly in the lower lobes (Fig. 1).

**Figure 2:**
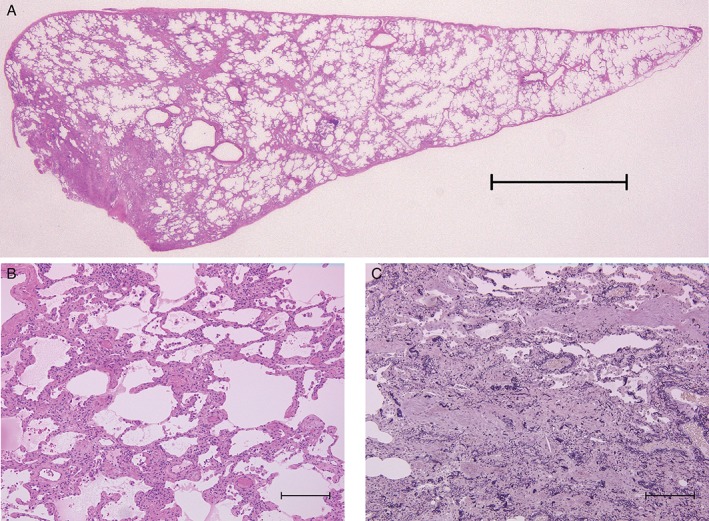
Pathological findings of the right lower lobe. (A) Haematoxylin–eosin stain. Low‐power view showing the inflammatory process following the original alveolar walls. These histological findings are characteristic of non‐specific interstitial pneumonia. The interlobular septum was thickened. Scale bar, 500 μm. (B) Haematoxylin–eosin stain. High‐power view showing interstitial inflammatory infiltration with mainly lymphocytes and plasma cells. Scale bar, 200 μm. (C) Elastica van Gieson stain. Occasional intraluminal organizations were found. Scale bar, 200 μm.

Bronchoalveolar lavage fluid was neither bloody nor milky. Transbronchial lung biopsy specimens were non‐diagnostic. We therefore performed surgical lung biopsy of the right lower lobe. Histological findings showed interstitial inflammation and interlobular septal thickening associated with inflammatory cell infiltration, mainly involving lymphocytes and plasma cells scantily associated with the intra‐luminal organization (Fig. 2). This was compatible with a diagnosis of cellular NSIP.

A skin biopsy showed oedematous changes and inflammatory cell infiltration, partly around the capillaries in the epidermis. Based on these findings, we diagnosed this case as NSIP associated with CADM. The patient was treated with prednisolone 30 mg/day (0.5 mg/kg/day) and cyclosporine 100 mg/day, with marked improvement of her chest CT findings. The dose of prednisolone was tapered to 5 mg/day, and she has remained stable for over a year.

## Discussion

The “crazy paving” appearance consists of scattered or diffuse ground‐glass attenuation superimposed on a network of linear opacities on thin‐section lung CT. This finding is non‐specific and has been described in a variety of diseases, including pulmonary alveolar proteinosis, diffuse alveolar damage superimposed on usual interstitial pneumonia, acute interstitial pneumonia, acute respiratory distress syndrome, *Pneumocystis jirovecii* pneumonia, drug‐induced pneumonitis, and cardiac pulmonary oedema 1, 2; however, it is extremely rare in NSIP 1, 2, 3. The common chest CT findings in patients with biopsy‐proven NSIP are lower lobe with peripherally predominant ground‐glass opacity with reticular abnormality, traction bronchiectasis and lower lobe volume loss. This patient thus represents a rare case of “crazy paving” appearance as the main CT finding in a patient with biopsy‐proven NSIP.

The linear network in the “crazy paving” appearance has been suggested to represent interlobular septal thickening, intralobular interstitial thickening, and airspace filling 1. Ground‐glass opacity may reflect the presence of airspaces or interstitial abnormalities, and if these pathological findings are slightly increased in severity and located at the borders of structures such as acini or secondary pulmonary lobules, they may be responsible for the network of linear opacities. Histopathological findings in our case showed interlobular septal thickening, which may have produced the linear network appearance. However, it was not possible to determine if these histological findings were located at the periphery of acini or secondary pulmonary lobules based on the biopsy specimens.

NSIP is often associated with connective tissue diseases, and it is therefore important to look for associated underlying diseases in patients presenting with NSIP. The current patient also had Gottron’s papules, which is the representative skin manifestation in dermatomyositis (DM); however, she had no signs of muscle weakness, and her creatine kinase level was within the normal reference range. These clinical findings indicated a clinical diagnosis of CADM. Anti‐melanoma differentiation‐associated gene 5 (MDA‐5) antibodies are strongly associated with the development of rapidly progressive interstitial lung disease (ILD) in patients with CADM, but our patient was negative for MDA‐5 antibodies. Ikeda et al. reported that the clinical features of anti‐MDA‐5 antibody‐negative CADM‐ILD was similar to those of classical DM‐ILD 4. Furthermore, the development of myositis may lag behind the onset of skin manifestations in some patients with DM, and continuous monitoring of muscle and other clinical symptoms is thus required. About 48% of patients with polymyositis/DM‐ILD are positive for anti‐ARS antibodies, and these patients have a better prognosis than those without anti‐ARS antibodies 5. The current patient was positive for anti‐ARS antibodies and responded well to prednisolone and cyclosporine therapy.

In conclusion, ILD with NSIP should be included in the differential diagnosis for patients presenting with a “crazy paving” appearance on thin‐section lung CT. Detailed clinical, radiological, pathological, and microbiological examinations are needed to exclude other diseases.

### Disclosure Statement

Appropriate written informed consent was obtained for the publication of this case report and accompanying images.
